# Depressed Mood Prediction of Elderly People with a Wearable Band

**DOI:** 10.3390/s22114174

**Published:** 2022-05-31

**Authors:** Jinyoung Choi, Soomin Lee, Seonyoung Kim, Dongil Kim, Hyungshin Kim

**Affiliations:** Department of Computer Engineering, Chungnam National University, Daejeon 34134, Korea; jinyoung00@cnu.ac.kr (J.C.); sxxmin.lee@g.cnu.ac.kr (S.L.); 1020peace@gmail.com (S.K.); dkim@cnu.ac.kr (D.K.)

**Keywords:** depressed mood, wearable band, unobtrusive monitoring, elderly depression

## Abstract

Depression in the elderly is an important social issue considering the population aging of the world. In particular, elderly living alone who has narrowed social relationship due to bereavement and retirement are more prone to be depressed. Long-term depressed mood can be a precursor to eventual depression as a disease. Our goal is how to predict the depressed mood of single household elderly from unobtrusive monitoring of their daily life. We have selected a wearable band with multiple sensors for monitoring elderly people. Depression questionnaire has been surveyed periodically to be used as the labels. Instead of working with depression patients, we recruited 14 single household elderly people from a nearby community. The wearable band provided daily activity and biometric data for 71 days. From the data, we generate a depressed mood prediction model. Multiple features from the collected sensor data are exploited for model generation. One general model is generated to be used as the baseline for the initial model deployment. Personal models are also generated for model refinement. The general model has a high recall of 80% in an MLP model. Individual models achieved an average recall of 82.7%. In this study, we have demonstrated that we can generate depressed mood prediction models with data collected from real daily living. Our work has shown the feasibility of using a wearable band as an unobtrusive depression monitoring sensor even for elderly people.

## 1. Introduction

Depression of the elderly is an important social problem to be solved. In 2020, South Korea ranked as the first among Organization for Economic Co-operation and Development (OECD) countries in number of depression cases [1]. But only 20% of mental patients visit hospital [2]. This is because most people do not think that they have a mental illness [3]. According to the Ministry of Health and Welfare in South Korea, 30.2% of the elderly living alone has been diagnosed with depression while 16.4% living with a spouse and 21.7% living with children have been diagnosed as depressed [4]. The Korea Institute for Health and Social Affairs reported deterioration of social relationships and isolation as the main causes of weakening the mental health of the elderly [5]. As the proportion of elderly people living alone is high, a depression monitoring system especially for elderly people living alone is in high demand.

Depression is an emotion that requires long-term monitoring, and it is important to observe features related to depression symptoms in daily life. Symptoms of depression include sleep changes, loss of interest or pleasure in activities, fatigue or loss of energy, feelings of worthlessness, depressed mood, weight change, etc. [6]. In this study, we decide to monitor the activity level and heart rate data to detect the amount of physical activity. On the other hands, other studies analyzed depression from visited location information, and type of activities [7,8]. Sleep time and light exposure are used as major features to recognize depression [9]. Many studies have been conducted to predict depression using sleep time with a wearable band [10,11] because of the convenience.

Studies using passive infrared (PIR) motion sensors in residential spaces to collect data from real living spaces are reported [12]. Individual depression can be predicted from daily life activities of spatial characteristics such as bedroom, bathroom, and kitchen. An attempt was also made to predict depression from physical characteristics such as voice and facial expression. Depressed mood has been associated with biological data such as facial expressions, voice and spoken words [13,14].

However, there are limitations in those approaches when we apply them to the elderly. Cameras and microphones may intrude users’ privacy and can lead to a sense of refusal. Monitoring method for the elderly should be performed in unobtrusive manner without privacy issues [15,16]. Elderly people do not easily accept complex equipment and experimental environments [17]. We must devise a natural and acceptable method which do not interfere their daily lives. Instead of utilizing multiple and complex devices, we chose a wrist band with multiple sensors integrated on it. We chose a E4 wearable band from Empatica [18] as our sensing device. It has photoplethysmogram (PPG), accelerometer (ACC), electrodermal activity (EDA), and temperature sensor within a device so that they can provide variety of biometric data for analysis.The other work used E4 wearable bands and smartphones to predict depression [19,20]. Those previous works explored features such as EDA sensor data, sleep characteristics, physiological data, and smartphone usage. However, we excluded sleep patterns and smartphone-based features to minimize the discomfort of the elderly. For data collection in residential spaces, it is important to minimize the feeling of being forced to do so [21]. We have the participants wear the band only while they are awake and at home.

In this paper, we propose to develop a model that predicts the depressed mood of the elderly living alone. We recruit 14 elderly from our neighboring community, not the patients registered at the medical institution. We want to predict if a person is either in a *normal* state or in a *mild depression* state with a wearable band. Since we did not generate our prediction model with patients, we focus on the depressed mood instead of depression as a disease.

We develop depressed mood prediction models from participants’ daily activity and biometric data. Data collection from the band has been performed for 71 days. Depression questionnaire has been surveyed once a week to be used as the labels. We want to see that the sensors on the E4 band can provide correlation to depressed mood. We select the stationary state, the signal cycle of the heart rate sensor, and biometric statistics as the features. We assume these features are correlated with the lose of interest or pleasure in activities, fatigue or loss of energy, feelings of worthlessness, and depressed mood symptoms. A general model for all the participants is generated to be used as the baseline model for the initial model deployment. Personal models are also generated for further refinement. The general model use 8 machine learning models and the personal models use the random forest model. The general model has a high recall of 80% in the k-NN model. Individual models achieved an average recall of 82.7%.

Our work is unique in that we focus on the single household elderly people. From our knowledge, no previous studies generated prediction model with single household elderly. Our depressed mood monitoring method is a feasible approach which do not interfere with the elderly people’s daily life. Our work has an advantage over previous studies since our work only use indirect user data instead of using privacy-sensitive direct user data. The contributions of this paper are as follows.

- First, we extracted features from the E4 wearable band related to the depression symptoms based on DSM-5. PPG, ACC, EDA, and temperature sensor data are related to depressed mood, fatigue or loss of energy, thoughts of death or suicidal ideation, and change in weight appetite symptoms. We analyzed the activity level and biometric features with the depression symptoms. Our work has an advantage over previous studies in that we work only with the indirect user data instead of privacy-sensitive direct user data.
- Second, the proposed models predict the general elderly people’s depressed mood, not patients. We collected data from 14 general single-household elderly people. Patient health questionnaire-9 (PHQ-9) and Korean version of the short form of geriatric depression scale-K (SGDS-K) depression questionnaires are surveyed as labeling the depressed mood. We predict depressed mood in two classes. We predict whether the elderly people are in the *normal* or the *mild depression*.
- Third, a general model and personal models are generated. We applied eight different machine learning models for the general model. The k-NN model performs the highest recall rate 80.0%. The personal model generated in three different modes such as ACC-Only Model, the PPG-Only Model and the ACC and PPG Model. The ACC-Only Model performs the highest recall rate 82.7% in average. These values are competitive with previous works using bio sensor data from a wearable band.

The remainder of this paper is organized as follows. Section 2 describes related research. The generation of the depressed mood prediction model is described in Section 3. Section 4 explains the process of collecting data. The prediction results of the models are presented in Section 5. Section 6 concludes this paper.

## 2. Related Works

To diagnose depression, psychopathological researches are typically based on clinical interviews or questionnaires from patients. These surveys are quantified as scores and are used as labels in most studies. The most reliable and widely used scales include the GDS-30 [22], Beck Depression Inventory [23] and PHQ-9 [24]. These surveys consist of a minimum of 9 items and a maximum of 21 items. Surveys can be subjective depending on individual feelings and circumstances. Even in a professional survey, the patient’s answers may not be trusted. Depression is known to be difficult even a doctor to diagnose. Long-term treatment and continuous surveys are needed for accurate diagnosis. However, it is not easy for the elderly to visit a hospital regularly.

Several attempts have been made to detect depression or depressed mood indirectly or automatically. Depressed emotions are related to sleep patterns and daily activity patterns. Activities of daily living (ADL) have been used to track physical activities related to psychological state [25,26,27,28]. They tried to monitor depression symptoms from various sources such as household appliance usage, biometric data, and electricity usage in combination. The main depression symptom is activity level, and the higher the depression, the lower the activity level. To analyze the trend of their activity, if the participant was a student, characteristics related to depression symptoms were extracted from everyday life, such as smartphone usage and moving distance between buildings [8]. From monitoring with a smartphone, they can distinguish activities such as walking, running, and sleeping from the sensors on the smartphone. This indicates the possibility that abstract states such as depression symptoms lassitude, anhedonia, and psychomotor retardation can also be inferred using a smartphone [29].

Another major depression symptom is sleep, which means that the depression state worsens when sleep duration is reduced. A wearable band-type device was also used to analyze the correlation between sleep and activity levels and depression [30,31]. It was mainly used with smartphones or other sensors rather than a single wearable band device. Smartphone usage, conversation pattern, location, sleep time, and heart rate were selected as common characteristics. It has been reported that body temperature is the most related characteristic of sleep [30]. As wearable devices are commercialized, accelerometer sensor and heart rate sensor data are also recognized as sufficient equipment to extract the characteristics of depression symptoms. Studies have also been attempted to predict depression by subdividing sleep patterns, such as classifying not only total sleep time but also rem sleep time or specific sleep time. In Japan, the characteristics related to 13 types of sleep patterns were extracted from general business people, and the results showed an accuracy of 63.9% and a recall of 63.9% [10]. Another research team showed the results that biometric data measured from 4 am to 6 am is the best feature for predicting depression. The research team predicted depression using step count and bpm, and showed results with 80% accuracy and 82% sensitivity [11].

There are several previous works that have applied E4 wearable bands. In the paper [32,33], the E4 band has been used to predict arousal which is sensitive to major depressive disorders. Paper [34,35] showed EDA and heart rate can be used for stress. The other work [19,20] is similar to our work in that they try to predict depression with the band. In the paper [32], the authors classified the arousal levels using EDA. Another paper [33] reported students correctly performed highly demanding tasks with a higher EDA. Their target is student pilots based on the arousal changes. Our work is different in that we predicting the depressed mood of elderly people living alone. In the paper [34], they observed significant differences in EDA and HR between different stress levels of games. The slow EDA signal reflects the gross effects. In the other paper [35], they analyzed the skin conductance level and heart rate to monitor stress recovery. They observed the effects of the acoustic environment on the experimenters’ anxiety and stress states. Anxiety or stress might affect the depressed mood. However, our goal is to monitor depressed mood. In the paper [19], the author showed the efficacy of machine learning techniques for predicting depression with EDA and acceleration. They combined the E4 wearable band sensor data and the smartphone information. Another paper [20], also used EDA, acceleration, and smartphones to predict depression.

Those previous works explored features such as EDA sensor data, sleep characteristics, physiological data, and smartphone usage. However, we excluded sleep patterns and smartphone-based features to minimize the discomfort of the elderly. Our prediction models are generated from ordinary elderly people while the previous works recruited depression diagnosed patients. We had challenges in the elderly people’s concern and inconvenience. We conducted a experiment with only E4 wearable band indoors unlike previous works.

A study was also conducted to determine an individual’s depressed state based on audio and text such as voice through interviews [14,36,37]. The pitch and strength of the voice signal were extracted by focusing on the prosody pattern of the voice signal. To analyze facial expressions together, facial landmarks were numbered to track facial expression changes according to the depressed state [37]. Another study was conducted that could immediately diagnose a depressed state based on the characteristics of synthesized speech and used words [38]. A study by psychologists found that depressed people had fewer facial movements. By automatically tracking facial features, parameters such as average duration and total duration were calculated to automatically analyze depression [39].

Most of the studies used multi-devices to characterize various depression symptoms. Most of the subjects in previous studies are depression diagnosed patients or included a variety of age groups. The purpose of our work is to analyze the depression symptoms with a minimum of a single device in order to target the elderly, who are the vulnerable groups. The elderly are sensitive to personal information and have a sense of objection to devices, with particular emphasis on minimizing the objection to multiple sensors. Therefore, a method of collecting biometric data from a wrist-attached band that does not interfere with daily life was proposed. Biometric data is data that has a deep relationship with emotions and has high reliability even in existing studies. The activity and biometric information were selected as major depression symptoms from accelerometer data and heart rate data.

Previous studies on predicting or estimating depression are summarized in Table 1. Although the results of each study show satisfactory performance, personal information exposure occurs due to the use of multiple devices. Social characteristics such as location and smartphone usage time were extracted. Individual emotional changes were inferred from voice and conversational information. In this study, we collected data from simple sensors such as ACC and PPG. We did not use privacy-sensitive data for the model generation. Concerns about personal information exposure were reduced by using biometric information rather than a camera, voice, and other direct personal information. Our prediction model showed a very competitive accuracy with the others. Our work focused on single-household elderly people so that we could analyze the relationship between daily activity and depression of elderly people.

## 3. Depressed Mood Prediction

The purpose of this study is to predict depressed mood from multiple sensor data. A general model and a personal model with a E4 wearable band are developed. This chapter presents the four steps of our model development.

### 3.1. Procedure of the Depressed Mood Prediction Modeling

**Figure 1:**
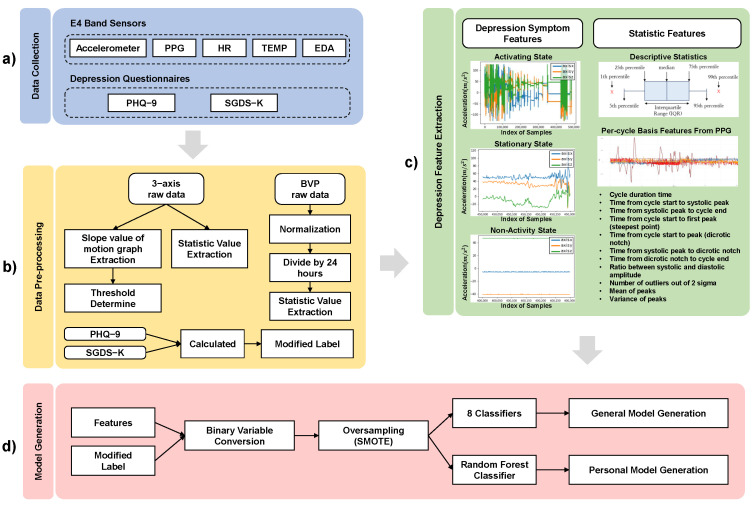
The model generation process proceeds sequentially from (**a**) to (**d**). (**a**) refers to collected biometric sensor data and questionnaire data. In (**b**), it refers to the pre-processing of the data collected in (**a**). The 3-axis accelerometer sensor data, BVP data, and questionnaire data, which are raw data of each sensor, are applicable. In (**c**), two types of features are extracted from data that has undergone pre-processing. These include features related to depression symptoms and statistical features. The general model and the personal model are generated from features and modified labels as shown in (**d**).

We develop the depressed mood prediction models through four steps. We predict the elderly people’s depressed mood whether they are in a *normal* state or a mild depressed mood. We generate two different prediction models. We generate a general model as a baseline for the initial model deployment. We generate personal models for model refinement. The four steps are data collection, pre-processing, feature extraction, and model generation. Figure 1 shows the procedure to generate the prediction models. The data collection step is collecting the E4 wristband sensor data and the depression questionnaire data. The pre-processing step is modifying the collected data into the format of the dataset required for the feature extraction step. The depression feature extraction step is extracting features related to depression symptoms. The model generation step is generating the general model and the personal models.

### 3.2. Data Pre-Processing

As the first step, the sensing data from the E4 wearable band and the depression questionnaire data are collected as shown in Figure 1. The E4 band has PPG, ACC, EDA, and temperature sensors (TEMP). Depression questionnaire data is the score of PHQ-9 questionnaire and the SGDS-K questionnaire. Numerical scores are mapped to extracted features as labels.

In Data Pre-processing, the pre-processing is modifying the collected raw data and survey data. The E4 watch generates more than one file every day. We need one representative feature value per day to be labeled with a depression score. Therefore, a process of merging multiple files into a daily file is required. When the E4 band is worn in the wrong position or too loosely, the recorded data might be abnormal values. Also the unknown data might be sensed when the wristband is turned on without being worn on the wrist. We remove the unknown data that occurs in these situations. Heart Rate (HR) data is used to eliminate these error intervals. The HR data files are calculated from the Blood Volume Pressure (BVP) data. If no pulse is detected for a certain period of time, the file is not generated. The error section is removed based on a specific threshold using the difference in the slope of the HR index value.

One quantified depressive state value is needed to be labeled. The PHQ-9 survey scores 0 to 27 points, and the GDS-K geriatric depression survey scores 0 to 15 points, and the results are calculated as quantitative values. The individual SGDS-K score of each participant was multiplied by the PHQ-9 score and used as a weight.

### 3.3. Feature Extraction

According to DSM-5 [6], symptoms of depression are sleep changes, loss of interest or pleasure in activities, fatigue or loss of energy, feelings of worthlessness, depressed mood, weight change, and suicide ideation. We related the sensors from the E4 wearable band with the symptoms described in DSM-5. Various sensor data are extracted from the E4 wearable band as shown in Table 2.

We extract sensor data that may contain depression symptoms from the sensors on E4 band. In previous studies [25,26,27,28], they defined physical activities as step count, using toilet, transferring (moving from a bed to a chair), housework, and jogging. In their work, authors related physical activities to the mood. However, our proposed experimental environment is indoors so that we simplified activities during our study.

In our study, to detect the amount of physical activity, we monitor the activity level and heart rate data. In the paper [41,42], authors reported when people are at stationary state, they are likely to be in depressed mood. We related the accelerometer sensor data with the fatigue or loss of energy which are the depressive symptoms defined in DSM-5. We extracted Moving/Stationary state features. The stationary state means no change in movement in a sitting state. We assume that as long as the elderly maintain the stationary state, it would be that they might lose of interest or pleasure in activities, fatigue or loss of energy, feelings of worthlessness, and depressed mood. The stationary state represents that there is no change in movement in a sitting state. For example, activities such as getting up from a seated state or clapping from a seated state are defined as not being stationary. If the state of being stationary without specific movement in the room continues, it interprets that the classifying the states could imply loss of interest or pleasure in activities, fatigue or loss of energy, and feelings of worthlessness.

Heart rate features are in the time domain and statistics data which are related to the diagnostic symptoms of depression. Heart rate variability refers to fluctuations in heart rate, which are controlled by the activity of sympathetic and parasympathetic nerves [43]. In previous studies, it has been reported that heart rate variability is lower in the case of major depressive disorder than in normal people, and it has been used as a biomarker. When crying, anxious, or angry, the sympathetic nerve is stimulated [44]. Most of the studies have extracted indicators from the time domain, which has high reliability and can be analyzed in a short time.

In the EDA sensor and TEMP sensor, descriptive statistics such as outlier ratio, mean, and standard deviation including coefficient of variation and outlier feature values are extracted and used to analyze depressed mood. All of the features are processed as aggregated representative values for one day and labeled with depression questionnaire values. Descriptive statistics such as expected values, 1st to 3rd quartiles, mean, and variance are used as features on a daily basis. Through the BVP sensor, more than 90 features are extracted from the signal data, including outliers detected based on the systolic and diastolic periods occurring within the signal cycle, and used as input data. Centering tendency, spreading degree, distribution shape, and symmetry degree values are extracted from the BVP values.

After features are calculated, the general model and personal models are generated in phase (d) Model Generation. The general model predicts depressed mood for all users. The personal models predict each elderly people’s depressed mood. To solve the data imbalance, the SMOTE algorithm is applied. We employed eight classification models for modeling the general model. For the personal model, a random forest model is used.

### 3.4. Depressed Mood Prediction Model Generation

**Figure 2:**
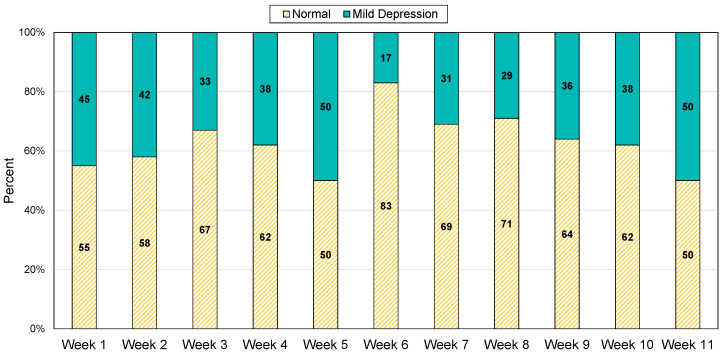
Weekly Ratio of Participants between Normal/Mild Depression Class.

The models we proposed predict whether the participant is in a *normal* state or a *mild depression*. The depressed mood is classified based on Formula (1). We go through several experiences to find out the threshold value of classifying the depressed mood states. The achieved threshold value has the highest accuracy, f1-score, and recall value for all the participants. The value of k that derives the best results is 0.5, and when the corresponding threshold is set, *normal* and *mild depression* are present in a ratio of approximately 2:1. Figure 2 is a graph showing weekly ratio of participants in the two classes. The weekly ratio of *normal* and *mild depression* for all participants is 1.9:1 in average.

(1) threshold=E(PHQscore×SGDSKscore)+k×σ(PHQscore×SGDSKscore)

A general model is developed to use as an objective indicator. Due to the limited training data, we focused to adopt a machine learning model instead of deep learning. Eight classification algorithms are applied for the model generation: K-NN classifier (KNN), Support Vector Machine (SVM), Decision Tree (DT), Random Forest (RF), Gradient Boosting (GB), XGBoost (XGB), 3- Multi-layer Perceptron (MLP), 4-MLP.

For personal modeling, three models are constructed by ACC sensor (ACC-Only Model), PPG sensor (PPG-Only Model), and both sensors together (ACC and PPG Model). A random forest model was applied to each model. The train data of the ACC sensor consists of 154 statistical features and two motion features. The train data of the PPG sensor consists of frequency domain features related to emotion. ACC and PPG Model is generated with all the features from the two sensors.

We evaluate the predictive models that are divided into the *“normal”* group and *“mild depression”* group. The k-fold cross-validation is used as an alternative to performance evaluation with the testing set. In the general model, k is set to five for the whole subject data. In the personal model, k is set to three for each subject. For the general model, the training and testing data were divided in a balanced manner from a stratified split.

## 4. Data Collection

This section describes the sensing plan for detecting depression in elderly people living alone. Section 3.1 describes the process performed to recruit the elderly that are living alone, and Section 3.2 describes the E4 wearable band sensing data. Section 3.3 explains the depression questionnaires. This study is approved by the Institutional Review Board at Chungnam National University (IRB approval number: 201907-SB-099-01).

### 4.1. Recruiting Elderly People Living Alone

We recruit 14 elderly people aged 65 or older living alone, who are registered at a local social welfare center. We go through a number of screening procedures to select final participants who are more likely to be in a depressed state. The selection process is mainly based on the interview and PHQ-9 and SGDS-K survey. The first, 48 elderly people are recommended by the welfare center. We visit each house with a social worker and explain the experiment in detail. Some of the elderly people refuse to participate in the experiment because they are reluctant to experiment with strangers. They also mention that the experiment is an invasion of their daily life. 23 elderly people voluntarily remain to continue in this house visit.

When we explain the detailed plan with the wearable band, some of them do not like the idea of wearing a band during their daily living. Nine out of 23 refuse to participate in the study because of this. Finally, 14 single household elderly agree to participate in our research. Two of them have been diagnosed with depression in the past years. One of them take occasional sedatives, and the other takes antidepressants.

The 14 participants consist of 12 females and 2 males. The average age is 76, and the oldest one is 86. All of them are attending special activities classes at welfare centers. They confirm that there is no physical discomfort in carrying out daily activities, and there are no abnormalities in cognitive function through the simple screening test for dementia. In addition, the following information is collected through a brief interview. Ten participants answer that they have a religion. Regarding the interaction between families, two of the participants regularly meet with their families. Looking at the living patterns of the participants, nine people live in apartments, three in the houses, and two in townhouses. Their apartments are small size having one bedroom and one bathroom.

### 4.2. E4 Sensor Data

Empatica E4 wearable bands are provided to the recruited participants. They wear the band every day while they stay at home. They are instructed not to wear it when go outside. Participants may remove the band when they work with water or when they sleeping. Recharging the band also requires untying the band. The sensing data is stored in the memory within the band. In order to dump the stored data and survey the depression questionnaires, we have a weekly meeting with the elders.

Participants wear the E4 band for 67 h a week on average. Four of them wear the band for more than 100 h, and 1 person for less than 30 h. The E4 band is a wearable device developed for research and it does not include a screen. It contains a photoplethysmogram (PPG), accelerometer (ACC), electrodermal activity (EDA), and temperature (TEMP) sensors. Its memory stores about 60 h of data in .csv format files. The PPG sensor uses light to measure oxygen saturation and blood volume changes in the bloodstream. The BVP file is the raw data of the PPG sensor, and Inter-Beat Interval (IBI) is the time interval between individual beats of the heart. The IBI sequence is obtained from the processing of the PPG/BVP signal, with an algorithm that already removes incorrect peaks due to noise in the BVP signal. The data files from the E4 band are as follows:

- ACC.csv—Data from 3-axis acceleometer sensor in the range [−2 g, 2 g] (sampled at 32 Hz);
- BVP.csv—Data from photoplethysmograph (PPG) (sampled at 64 Hz);
- EDA.csv—Data from the electrodermal activity sensor in μS (sampled at 4 Hz);
- IBI.csv—Inter beat intervals (intermittent output with 1/64 s resolution);
- TEMP.csv—Data from temperature sensor expressed in degrees on the Celsius (${}^{\circ }$C) scale (sampled at 4 Hz);
- HR.csv—This file contains the average heart rate values, computed in spans of 10 s.

Empatica provides an API that can extract heart rate variability (HRV), which is affected not only by physical activity but also by changes in the sympathetic and parasympathetic nervous systems that are closely related to emotions. Major features can be extracted in the time domain, such as the distance between peaks and the slope [45,46]. The parasympathetic nervous system is activated when we are happy or unhappy, and the sympathetic nervous system is affected when we are nervous, anxious, or anxious. In this study, we select features based on heart rate cycles in the time domain [47]. ACC sensors are expected to provide activity information of the elders. Various features from the ACC sensors are extracted to find a correlation with the elder’s daily activity.

### 4.3. Depression Assessment Questionnaire

The participants surveyed a Korean version of PHQ-9 questionnaire and the SGDS-K questionnaire. The PHQ-9 is conducted once a week throughout the experiment. In addition, SGDS-K is performed once at the beginning of the experiment to determine the existing degree of depression. SGDS-K is also used as a participant selection criterion but also is used as a weighting factor for labeling. PHQ-9 total score for each elderly is used as the label for the features extracted from the sensor data. Although both surveys are self-administered questionnaires, it is difficult for most of the participants to conduct the survey by themselves, due to their presbyopia. Therefore, we perform the survey with them. On days when they can not meet due to personal circumstances, the participants conduct the survey by themselves and have them bring it to the next meeting day with us.

The SGDS-K consists of 15 questions, and the answers are yes and no, and if yes, 1 point is counted. The highest score is 15, and if the score is 6 or higher, consultation with a specialist is recommended. Through the results of the SGDS-K, it is possible to examine whether the proportions of depressed, *normal*, and *mild depression* among all the participants are evenly distributed. PHQ-9 is composed of 9 items that correspond to the diagnostic criteria for major depressive disorders in DSM-5 (Diagnostic and Statistical Manual of Mental Disorders). The total score of the PHQ-9 is used for evaluating the severity of depression. It ranges from 0–27 points, reflecting the higher the severity of depression as the score increases [48,49].

## 5. Results

This study describes the results of depression model generation described in the previous chapter. For 71 days, 14 senior citizens living alone are recruited and participated in the experiment to generate the model. E4 wearable bands are distributed and biometric data are collected from the smart band while they are at home. The results of a conducted survey are explained. The data collection and depression model prediction results are explained.

### 5.1. Depression Survey Results

**Figure 3:**
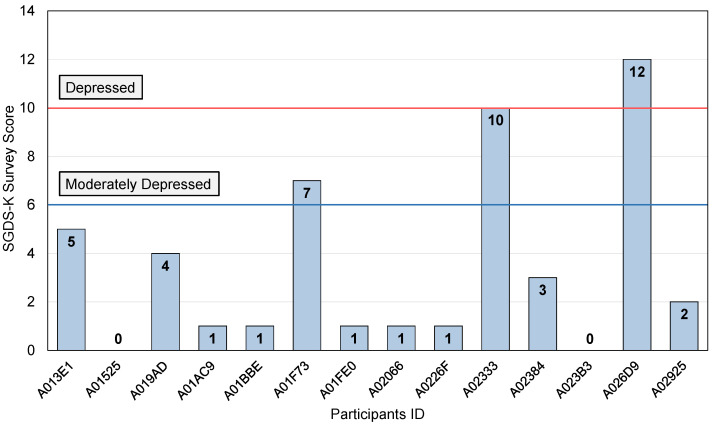
Results of SGDS-K Score for All Participants.

In this study, SGDS-K and PHQ-9 depression questionnaire scales are asked for the elderly living alone in Korea. SGDS-K is performed once before the beginning of the data collection. Figure 3 shows the results of SGDS-K for all the experiment participants. The average score of 14 participants is 3, the lowest score is 0. The highest score is 12. Two participants who scored 12 and 10 can be diagnosed with depression. Participant A01F73, who scored between 6 and 9, can be diagnosed with severe depression. The remaining 11 participants are diagnosed as normal. However, this survey was surveyed only once at the beginning of the experiment, indicating the depression state at the time.

**Figure 4:**
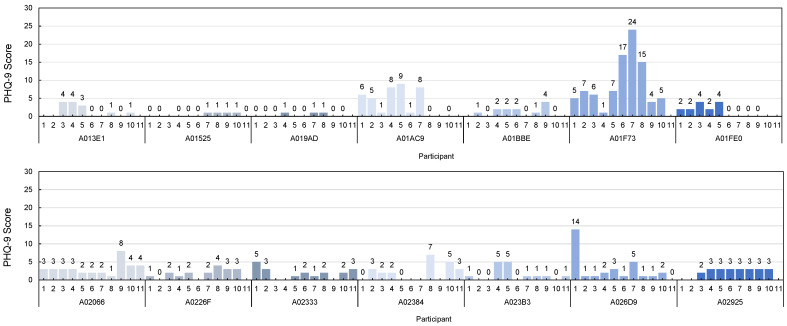
Results of Weekly PHQ-9 Score for All Participants.

PHQ-9 is performed on all the participants once a week for about 10 weeks. The survey is conducted every Wednesday and Friday. As shown in the Figure 4, the largest variation of individual weekly scores is 23 points. In this case, there is an obituary of the participant’s brother during the experiment period. When this patient conducts the PHQ-9 after the funeral, the total score is 24. This score is the highest score among all survey results. This score can be interpreted as severe depression. The person with the next greatest change has a difference of 14 points. The participant frequently mentioned concerns about his son, especially during the experiment. This participant’s highest PHQ-9 score is 14, which could be diagnosed as severe depression. The third most varied person has a 9-point difference. This participant suffers from health problems such as dental care and back pain during the experiment period.

The PHQ-9 questionnaire is typically classified into five levels of depression. When the total score is within 0∼4, it is a normal range, indicating a calm state without depression. A score of 5 to 9 indicates that the degree of depression is mild and requires follow-up. A score of 10∼14 indicates that the depressive condition is moderate in severity and should be considered for treatment. A score of 15∼19 is moderate and requires medication or counseling. A score of 20∼27 is severe and requires aggressive and psychiatric treatment. The average of the total PHQ questionnaire scores collected during the entire experimental period is 3 (std = 2.56), the lowest mean score is 0 (std = 0.61), and the highest mean score is 9 (std = 5.81). A score of 0 is in the normal category and a score of 9 represents *mild depression*. During the experiment period, the average change of the participant’s depression is about 4 points (std = 5.72). At the distribution of the number of responses at the PHQ-9 survey, most responses are in the minimal depression category. However, as the SGDS-K survey results, 10 participants have *mild depression* or *severe depression*, we modified the PHQ-9 survey score combining with the SGDS-K survey score to predict the depressed mood as *normal* and *mild depression*.

### 5.2. General Model Generation

A general model is constructed from the E4 data of all participants. More than 90 features are extracted, including the coefficient of variation for the ACC sensor data, EDA data, BVP data, HR data, and body temperature data. The activity level of the body is extracted from ACC sensors as total sum acceleration (*S_TOT_*) which is signal vector magnitude in Equation (2) [50,51].

(2) $$S_{TOT}=\sqrt{{\left(A_{x}\right)}^{2}+{\left(A_{y}\right)}^{2}+{\left(A_{z}\right)}^{2}}\;$$

The value of *S_TOT_* is 1 g, which is equal to the acceleration of gravity when the body is at rest. Changes in acceleration values on the x, y, and z axis can be calculated to infer changes in physical activity. The coefficient of variation, the correlation coefficient between two axis, and *S_TOT_* are features with high explanatory power when classifying daily activities from the accelerometer sensor. In addition, to verify the proof of the concept for the PHQ survey, 15 labels are set by modifying the PHQ score and the SGDS-K score.

Eight classification algorithms are applied for the model generation; KNN, SVM, DT, RF, GBC, XGB, 3-MLP, and 4-MLP. Since the class imbalances, The model was trained after oversampling because of the class imbalances The accuracy, recall, and f1-score results of each model are shown in the Table 3. The model that showed high accuracy and recall values was the KNN. Models with an accuracy of 60% or more are sequentially RF, GBC, 4-MLP, XGB, and 3-MLP. Both high recall and accuracy values are achieved with the 4-MLP model. The depression surveys score values are normalized from the PHQ results with SGDS-K score as a weighting factor. The recall value was 80% in the KNN model and 78% in the 4-MLP model.

### 5.3. Personal Model Generation

For personal modeling, three models are constructed by ACC sensor (ACC-Only Model), PPG sensor (PPG-Only Model), and both sensors together (ACC and PPG Model). The train data of the ACC sensor consists of 154 statistical features and 2 motion features. The train data of the PPG sensor consists of four frequency features related to emotion. ACC and PPG Model is generated with all the features from the two sensors. The individual answers can be subjective, the surveyed PHQ scores need to be normalized to be used as the training label. This is also related to the participants’ characteristics and their life attitudes. As the SGDS-K survey results, 10 participants have *mild depression* or *severe depression*, we modified the PHQ-9 survey score by combining the SGDS-K survey score to predict the depressed mood as *normal* and *mild depressed* mood.

The random forest model is used as the personal models. The personal models are generated for each participant as shown in Table 4. One participant’s model is excluded because the participant’s PHQ label has no variation at all during the experiment. The ACC-Only Model shows better performance than the PPG-Only model. This might mean that the ACC features have a higher correlation to depression. However, considering a number of features used for training, the PPG features have a much higher correlation to depression. This is attributed to the ACC and PPG Model. Accuracy, F1 score and recall values reflect that ACC features are better matched to the depression prediction. Recall has importance for the prediction of depression, and the performance is from 74.4% with PPG-Only Model up to 82.7% with ACC-Only Model. Among the 13 participants, participant A01BBE and A019AD show lower accuracy than others. This is due to the severe imbalance in the PHQ score. Also, the total number of sensed data of the two is far smaller than others. Though we tried to reduce data imbalance with SMOTE during model generation, if the original data is highly skewed, then the general model cannot overcome the problem. Participant A01F73 has relatively poorer performance with PPG-Only Model than others. This participant had his family funeral during the experiment and we suspect that severely affected his emotion so that PPG data as well. This may suggest that to detect depression with sensors, personal characteristic correlation to sensors should be considered.

## 6. Discussion and Conclusions

Elderly people living alone are more exposed to depression due to social isolation. Depression is an emotion that requires long-term observation and treatment. We propose a depressed mood prediction model for elderly people with a wearable band. This proposal has a novel approach by showing the feasibility of unobtrusive depression monitoring, especially for the general elderly people with a wearable band in daily life.

We recruited 14 ordinary elderly people without a history of depression diagnosis. We carried out depression surveys and collected sensor data from daily life for 71 days. Depression survey data were more biased toward normal status, so that we targeted to predict depressed mood to prevent worsening into depression. We intended to analyze the association of the sensors of the E4 wearable band with the symptoms of depression described in DSM-5.

Several studies tried to predict depression with E4 band [19,20]. Various features such as sleep patterns, motion, and biometric data were extracted. We excluded sleep patterns and smartphone-based features to minimize the discomfort of the elderly. Our work is different in that we collected data from ordinary elderly people while the previous works recruited depression diagnosed patients. We had challenges in the elderly people’s concern and inconvenience. Elderly people are hesitant to apply modern complex devices due to various reasons such as electromagnetic waves from devices and leakage of their privacy. They have low confidence in wearable devices. The recruited participants are characterized by living alone in a small space and aged between 67 to 86 years old. We conducted a experiment only with E4 wearable band and we monitored indoors unlike previous works.

In papers [41,42], authors reported when people are in a stationary state, they are likely to be in a depressed mood. We related the accelerometer sensor data with the fatigue or loss of energy which are the depressive symptoms defined in DSM-5. We extracted Moving/Stationary state features. The stationary state means no change in movement in a sitting state. Our work has shown the relationship between the stationary state and depressed mood.

A weekly PHQ-9 questionnaire is administered to observe changes in the depression state of the elderly during the experimental period. The SGDS-K questionnaire is used to verify the PHQ questionnaire labels. In the personal model, three modes based on the sensor data were observed. In general, activity sensors have a higher prediction accuracy than heart rate sensors. This represents that elderly people have better exposure to daily activities than their biometric features. As expected, the personal models have shown better accuracy and recall than the general model.

However, this proposal has its limitation of collecting certain data for the depressed participants. The participants have not been diagnosed with depression and have regular social activities. They might be in a good mood throughout the duration of the experiments. Also, the survey may not be trusted depending on the individual’s subjectivity and the situations at the time of the survey. This affected the PHQ-9 survey results to be more biased toward normal data while we tried to recognize the *“mild depression”* label as the depressed status. Although the depressed state data was not evenly distributed, this paper does not aim to diagnose depression in patients, so we could solve the problem. The general model and personal model are generated. The general model developed with 4-MLP has shown 69% accuracy and 78% recall and the k-NN model has shown 80% recall. The personal model is generated in three different modes by sensor data which are the ACC-Only Model, the PPG-Only Model and the ACC and PPG Model. The ACC-Only Model has shown the highest recall rate 82.7% in average. These values are competitive than previous studies which use wristband.

## Figures and Tables

**Table 1:** Summary of Related Works.

	Devices/Sensors	Privacy Level	Type of Subjects	Analysis Scale	Performance	
			Scope	Total #		
**Our Work**	**Wearable Device (E4 Wearable Band)**	**High**	**Non Patients (Aged 65 and Above)**	**14**	**2 Classes**	**Mean Recall in Individual Models 82.7%**
[19]	Wearable device (E4 wearable band), Smartphone	Low	Patients (20–73 years old)	12	Estimating modified survey scores	Root mean squared error 2.8
[40]	Pressure, Electrical, Contact Sensors	Low	Patients (All ages)	340	Comparing between different conditions	Adjusted odd ratio 0.85
[38]	Audio	Low	About half of the Patients included (23–69 years old)	59	2 Classes	Recall 69.5% to 71.0%
[20]	Wearable Device (E4 wearable band), Smartphone	Low	Patients (19–73 years old)	31	Estimating survey scores	Mean Absolute Error 3.88 to 4.74
[30]	Wearable device, ACC, HR, Skin temp., Ultraviolet light exposure	High	Patients (Aged 20 and above)	45	2 Classes	Accuracy 74%, Sensitivity 66%, Specificity 81%
[31]	Smartphone, HRV	Low	Non patients (24–68 years old)	60	2 Depression, Anxiety, Stress	Test Statistic Values (r-value, *p*-value)

**Table 2:** Correlation between Sensor Data and Depression Symptoms.

Sensor	E4 Wearable Band			
	PPG	ACC	EDA	TEMP
**Extracted Sensor Data**	Heart Rate Time Domain features Frequency Domain features	X, Y, Z axis statistic features Moving, Stationary states	Skin activity statistic features	Temperature statistic features
**Related Depression Symptoms**	Depressed Mood	Fatigue or Loss of energy	Thoughts of death or suicidal ideation	Change in weight or appetite

**Table 3:** Results of General Model with SMOTE.

Model	Accuracy	Recall	F1-Score
**k-NN**	67.0	80.0	61.0
**Support Vector**	57.0	30.0	25.0
**Decition Tree**	62.0	40.0	36.0
**Random Forest**	66.0	43.0	43.0
**Gradient Boost**	66.0	47.0	45.0
**XG Boost**	59.0	30.0	26.0
**3-MLP**	71.0	72.0	61.0
**4-MLP**	69.0	78.0	61.0

**Table 4:** Personal Model Results.

Subj. ID	ACC-Only			PPG-Only			ACC and PPG		
	Accuray	F1 Score	Recall	Accuray	F1 Score	Recall	Accuray	F1 Score	Recall
**A01F73**	68.1	84.2	80.6	43.1	36.5	47.2	55.6	46.7	72.2
**A01AC9**	66.7	64.8	80.0	66.7	61.8	50.0	69.4	69.0	55.6
**A02066**	72.5	78.5	85.0	54.2	53.8	60.0	72.5	78.5	85.0
**A01FE0**	90.1	84.6	94.4	84.5	88.6	89.7	79.8	84.3	95.2
**A0226F**	66.3	71.7	73.8	75.8	74.5	77.8	76.6	78.0	80.2
**A02333**	84.7	82.1	91.7	68.1	51.9	77.8	77.8	91.7	100
**A02384**	79.2	82.2	79.2	72.9	79.5	91.7	81.2	81.2	87.5
**A023B3**	94.6	93.3	93.0	82.8	85.9	85.9	89.8	89.8	93.3
**A01BBE**	57.5	51.9	50.0	57.5	64.7	73.3	52.5	56.7	56.7
**A019AD**	62.7	65.8	71.4	65.5	56.6	60.3	70.6	64.2	66.7
**A013E1**	94.2	87.8	100	84.7	83.6	88.9	94.2	94.3	96.3
**A01525**	78.2	70.0	76.2	77.8	74.2	65.1	80.6	74.5	75.4
**A026D9**	80.6	95.2	100	80.6	88.6	100	91.7	95.2	100
**Average**	76.6	77.9	82.7	70.3	69.2	74.4	76.3	77.2	78.5
